# Supplementary material for: Immune-mediated hookworm clearance and survival of a marine mammal decrease with warmer ocean temperatures
Source: eLife. 2018 Nov 6;7:e38432. doi: 10.7554/eLife.38432 (PMC6245726; doi:10.7554/eLife.38432)
Supplement: Supplementary file 1. [file elife-38432-supp1.docx]

Supplementary file 1. Selected binomial generalized linear mixed models for hookworm mortality in South American fur seal (*Arctocephalus australis*) pups. Models are ranked based on Akaike’s information criteria.

| Model | Predictors | df | logLik | AICc | ∆AIC | AIC weights | ^*^R^2^  _GLMM(m)_ | ^*^R^2^  _GLMM(c)_ |
| --- | --- | --- | --- | --- | --- | --- | --- | --- |
| 1 | BUN + HW Burden + Albumin + Infect. Per. + IgG + Glucose | 8 | -17.33 | 51.72 | 0.00 | 0.27 | 81.4 | 83.3 |
| 2 | BUN + HW Burden + Infectious Period + IgG + Glucose | 7 | -18.95 | 52.72 | 1.00 | 0.16 | 81 | 83.1 |
| 3 | BUN + HW Burden + Infectious Period + IgG + Glucose + HW Burden*Infectious Period | 8 | -17.98 | 53.01 | 1.29 | 0.14 | 82.3 | 83.4 |
| 4 | HW Burden + Infectious Period + IgG + Glucose + HW Burden: Glucose | 7 | -19.45 | 53.73 | 2.01 | 0.10 | 88.5 | 90.9 |
| 5 | BUN+ HW Burden + Infectious Period + IgG + Glucose + HW Burden: Glucose + Lymph. + Glob. + Albumin | 10 | -16.13 | 53.89 | 2.17 | 0.09 | 84.8 | 85.7 |
| 6 | BUN+ HW Burden + Infectious Period + IgG + Glucose + Lymph. + Albumin | 9 | -17.31 | 53.96 | 2.24 | 0.09 | 81.5 | 83.3 |
| 7 | HW Burden + Infectious Period + IgG + Glucose + HW Burden: Infectious Period | 7 | -20.29 | 55.39 | 3.67 | 0.04 | 80.1 | 82.5 |
| 8 | HW Burden + Infectious Period + IgG + Glucose | 6 | -21.40 | 55.40 | 3.68 | 0.04 | 78.8 | 82.2 |
| 9 | HW Burden + Infectious Period + IgG + Glucose + Lymph. + Albumin + BUN + Hb | 10 | -17.31 | 56.26 | 4.54 | 0.03 | 81.5 | 83.3 |
| 10 | HW Burden + Infectious Period + IgG + Glucose + Lymph. + Albumin + BUN + Hb+ Glob. | 11 | -16.17 | 56.33 | 4.61 | 0.03 | 81.5 | 83.3 |

BUN= Blood Urea Nitrogen, HW =Hookworm, Lymph.= Lymphocytes, IgG=Parasite (hookworm) specific IgG, Hb=Hemoglobin

^*^ Marginal and conditional pseudo R-squared values were calculated as described by Nakagawa and Schielzeth 2013.
