# Supplementary material for: Immune-mediated hookworm clearance and survival of a marine mammal decrease with warmer ocean temperatures
Source: eLife. 2018 Nov 6;7:e38432. doi: 10.7554/eLife.38432 (PMC6245726; doi:10.7554/eLife.38432)
Supplement: Supplementary file 2. [file elife-38432-supp2.docx]

Supplementary file 2. Multimodel coefficient estimates, standard errors (SE), Z and P values of predictors of hookworm mortality in South American fur seal pups. Reported values were extracted from the averaged top ranked models (∆AIC < 2.0) showed in supplementary table 1.

| Predictors | Estimate | SE | Z | P |
| --- | --- | --- | --- | --- |
| Intercept | -5.92 | 7.46 | 0.79 | 0.430 |
| HW Burden | 1.47 | 0.56 | 2.61 | 0.009 |
| IgG | -0.41 | 0.17 | 2.45 | 0.014 |
| Glucose | -0.59 | 0.37 | 1.60 | 0.109 |
| Infectious Period | 0.17 | 0.06 | 2.87 | 0.004 |
| BUN | -0.45 | 0.23 | 1.97 | 0.049 |
| Albumin | 0.19 | 0.20 | 0.93 | 0.354 |

HW = Hookworm, IgG= Parasite (hookworm) specific IgG, BUN= Blood Urea Nitrogen
