# Supplementary material for: Immune-mediated hookworm clearance and survival of a marine mammal decrease with warmer ocean temperatures
Source: eLife. 2018 Nov 6;7:e38432. doi: 10.7554/eLife.38432 (PMC6245726; doi:10.7554/eLife.38432)
Supplement: Supplementary file 3. [file elife-38432-supp3.docx]

Supplementary file 3. External factors affecting South American fur seal pup growth rate. Presented are several models ranked by second order Akaike’s information criteria.

| Model | Predictors | df | logLik | AICc | ∆AIC_c_ | AIC weight | ^*^R^2^ |
| --- | --- | --- | --- | --- | --- | --- | --- |
| 1 | Attendance + infected group (died) | 5 | -347.01 | 704.86 | 0.00 | 0.39 | 54.30 |
| 2 | Attendance + infected group (died) + Infectious period | 6 | -346.14 | 705.46 | 0.60 | 0.29 | 55.38 |
| 3 | Attendance + infected group (died) + Infectious period + HW burden | 7 | -345.62 | 706.83 | 1.97 | 0.15 | 55.97 |
| 4 | Attendance + infected group (died) + Sex (male) | 6 | -346.97 | 707.13 | 2.27 | 0.13 | 54.41 |
| 5 | Attendance + infected group (died) + Infectious period + HW burden + Sex (male) | 8 | -345.58 | 709.24 | 4.38 | 0.04 | 56.02 |
| 6 | Infected group (died) + Infectious period + HW burden + Sex (male) | 7 | -353.55 | 722.70 | 17.84 | 0.00 | 46.04 |
| 7 | Attendance + infected group (treated) + Infectious period + HW burden | 6 | -355.05 | 723.28 | 18.41 | 0.00 | 43.93 |
| 8 | Attendance + Infectious period + HW burden + Sex (male) | 6 | -355.65 | 724.49 | 19.63 | 0.00 | 43.05 |
| 9 | Attendance + infected group (treated) + Infectious period * HW burden | 7 | -354.79 | 725.17 | 20.31 | 0.00 | 44.30 |
| 10 | Attendance + infected group (treated) + Infectious period + HW burden + Sex (male) | 7 | -355.04 | 725.69 | 20.83 | 0.00 | 43.93 |

HW = Hookworm.
