# Supplementary material for: Immune-mediated hookworm clearance and survival of a marine mammal decrease with warmer ocean temperatures
Source: eLife. 2018 Nov 6;7:e38432. doi: 10.7554/eLife.38432 (PMC6245726; doi:10.7554/eLife.38432)
Supplement: Supplementary file 4. [file elife-38432-supp4.docx]

**Supplementary file 4**. Averaged coefficients, standard errors (SE), Z and P values of top ranked models for pup’s growth showed in supplementary table 3.

| Predictors | Coefficients | SE | Z | P |
| --- | --- | --- | --- | --- |
| Intercept | 3.8184 | 0.1 | 34.59 | 2.0 x10^-16^ |
| attendance | 0.0292 | 0.006 | 4.67 | 3.0 x10^-6^ |
| Group (Died) | -0.8743 | 0.28 | 3.039 | 0.00237 |
| Group (Treated) | -0.0149 | 0.21 | 0.069 | 0.94 |
| Infectious period | -0.0097 | 0.007 | 0.72 | 0.47 |
| Hookworm Burden | -0.0022 | 0.0012 | 0.32 | 0.74 |
