# Supplementary material for: Immune-mediated hookworm clearance and survival of a marine mammal decrease with warmer ocean temperatures
Source: eLife. 2018 Nov 6;7:e38432. doi: 10.7554/eLife.38432 (PMC6245726; doi:10.7554/eLife.38432)
Supplement: Supplementary file 5. [file elife-38432-supp5.docx]

**Supplementary file 5**. Negative binomial generalized linear models for CD3 lymphocytes response in South American fur seal pups infected with hookworms. Models are ranked based on second order Akaike information criteria (AIC_c_).

| Models | Predictors | df | logLik | AICc | ∆AIC_c_ | AIC weight | ^*^R^2^ |
| --- | --- | --- | --- | --- | --- | --- | --- |
| 1 | HW Burden + nursing + growth rate + growth rate : nursing | 6 | -243.1 | 499.4 | 0.00 | 0.49 | 89.14 |
| 2 | HW Burden + IgG + nursing + growth rate + growth rate * nursing | 7 | -242.9 | 501.4 | 1.99 | 0.18 | 89.22 |
| 3 | HW Burden + Lymphocytes + nursing + growth rate + growth rate * nursing | 7 | -243.0 | 501.7 | 2.23 | 0.16 | 89.17 |
| 4 | HW Burden + basophils + nursing + growth rate + growth rate * nursing | 7 | -243.0 | 501.7 | 2.29 | 0.16 | 89.18 |
| 5 | HW Burden + basophils + nursing + growth rate | 6 | -248.7 | 510.6 | 11.20 | 0.00 | 87.40 |
| 6 | HW Burden + lymphocytes + nursing + growth rate | 7 | -248.7 | 513.1 | 13.63 | 0.00 | 87.40 |
| 7 | HW Burden + basophils + IgG + nursing + growth rate + IgG* nursing | 8 | -248.5 | 515.1 | 15.70 | 0.00 | 87.47 |

HW= Hookworm, IgG= Parasite specific IgG

^*^R^2^= pseudo-R-squared value based on the likelihood-ratio test.
