# Supplementary material for: Immune-mediated hookworm clearance and survival of a marine mammal decrease with warmer ocean temperatures
Source: eLife. 2018 Nov 6;7:e38432. doi: 10.7554/eLife.38432 (PMC6245726; doi:10.7554/eLife.38432)
Supplement: Supplementary file 6. [file elife-38432-supp6.docx]

**Supplementary file 6**. Averaged coefficients, standard errors, Z and P values of top ranked models for CD3 lymphocytes response showed in supplementary table 5.

| Predictor | Coefficients | SE | Z | P |
| --- | --- | --- | --- | --- |
| (Intercept) | 0.6030724 | 0.2019958 | 2.934 | 0.00335 |
| Nursing | 0.0979182 | 0.0223123 | 4.312 | 1.62e-05 |
| Growth Rate | 0.039181 | 0.0034662 | 11.108 | < 2e-16 |
| Hookworm Burden | 0.009398 | 0.0036554 | 2.526 | 0.01152 |
| Growth Rate : Nursing | -0.0011898 | 0.0003226 | 3.624 | 0.00029 |
| Parasite Specific IgG | 0.000525 | 0.00171 | 0.303 | 0.76189 |
