# Supplementary material for: Immune-mediated hookworm clearance and survival of a marine mammal decrease with warmer ocean temperatures
Source: eLife. 2018 Nov 6;7:e38432. doi: 10.7554/eLife.38432 (PMC6245726; doi:10.7554/eLife.38432)
Supplement: Supplementary file 7. [file elife-38432-supp7.docx]

**Supplementary file 7**. Negative binomial generalized linear mixed models for hookworm infectious period in Osuth Americna fur seal pups. Models are ranked based on second order Akaike’s information criteria (AIC_c_)

| Model | Predictors | df | logLik | AICc | ∆AIC_c_ | AIC_c_ weight | R^2^_m_ | R^2^_c_ |
| --- | --- | --- | --- | --- | --- | --- | --- | --- |
| 1 | HG + HW Burden + IgG | 6 | -496.25 | 1005.10 | 0.00 | 0.34 | 74.2 | 80.4 |
| 2 | Globulins + HG + HW Burden + IgG | 7 | -495.69 | 1006.19 | 1.09 | 0.20 | 75.4 | 80.5 |
| 3 | Cholesterol + Globulins + HG + HW Burden + IgG | 8 | -495.25 | 1007.56 | 2.46 | 0.10 | 74.8 | 80.2 |
| 4 | Cholesterol + Globulins + Glucose + HG + HW Burden + IgG | 9 | -494.25 | 1007.84 | 2.74 | 0.09 | 72.8 | 79.8 |
| 5 | Globulins + HG + HW Burden + IgG | 8 | -495.64 | 1008.34 | 3.24 | 0.07 | 75.3 | 80.5 |
| 6 | Cholesterol + Globulins + Glucose + HG + HW Burden + IgG + Glucose : IgG | 10 | -493.35 | 1008.34 | 3.24 | 0.07 | 72 | 79.4 |
| 7 | Globulins + HG + HW Burden + IgG + Glucose : Cholesterol | 8 | -495.68 | 1008.42 | 3.32 | 0.07 | 75.4 | 80.4 |
| 8 | Cholesterol + Globulins + Glucose + HG + HW Burden + IgG + Glucose : Cholesterol | 10 | -493.47 | 1008.58 | 3.48 | 0.06 | 72.4 | 79.4 |
| 9 | Cholesterol + Globulins + HG + HW Burden + Glucose : IgG | 8 | -498.24 | 1013.54 | 8.44 | 0.01 | 73.3 | 80.8 |
| 10 | IgG | 4 | -508.34 | 1024.97 | 19.87 | 0.00 | 71.9 | 79.2 |

HG=hemoglobin, HW=hookworm, IgG=Parasite specific IgG

R^2^_m_= Marginal pseudo-R-squared calculated based on Nakagawa et al. 2017.

R^2^_c_=Conditional pseudo-R-squared calculated based on Nakagawa et al. 2017.
