# Supplementary material for: Immune-mediated hookworm clearance and survival of a marine mammal decrease with warmer ocean temperatures
Source: eLife. 2018 Nov 6;7:e38432. doi: 10.7554/eLife.38432 (PMC6245726; doi:10.7554/eLife.38432)
Supplement: Supplementary file 8. [file elife-38432-supp8.docx]

**Supplementary file 8**. Averaged coefficients, standard errors, Z and P-values of predictors for hookworm infectious period based on the top ranked models showed in supplementary table 7.

| Predictor | Coefficient | SE | Z | P |
| --- | --- | --- | --- | --- |
| Intercept | 3.635018 | 0.206354 | 17.47 | 2x10^-16^ |
| Parasite specific IgG | -0.017141 | 0.002598 | 6.542 | 2x10^-16^ |
| Hemoglobin | -0.032178 | 0.015306 | 2.084 | 0.03717 |
| Hookworm burden | 0.060301 | 0.021501 | 2.78 | 0.00543 |
| Total globulins | 0.001208 | 0.002453 | 0.49 | 0.62434 |
