# Supplementary material for: Immune-mediated hookworm clearance and survival of a marine mammal decrease with warmer ocean temperatures
Source: eLife. 2018 Nov 6;7:e38432. doi: 10.7554/eLife.38432 (PMC6245726; doi:10.7554/eLife.38432)
Supplement: Supplementary file 9. [file elife-38432-supp9.docx]

**Supplementary file 9**.

Hookworm prevalence, median hookworm burden, hookworm mortality and mean concentration of Chlorophyll-a and sea surface temperature (at December) during 10 South American fur seal reproductive seasons at Guafo Island, Southern Chile.

| Year | Hookworm prevalence (%) | Hookworm burden (number nematodes) | Hookworm Mortality (%) | Chlorophyll-a (mg/m^3^) | Sea Surface Temperature (^o^C) |
| --- | --- | --- | --- | --- | --- |
| 2005 | 100 | 450 | 33 | 1.8 | 13.2 |
| 2006 | 100 | 590 | 40 | 1.14 | 13.2 |
| 2007 | 81 | 210 | 13 | 7.33 | 12.3 |
| 2008 | 100 | 510 | 40 | 2.1 | 13.4 |
| 2012 | 100 | 570 | 42 | 0.83 | 13.9 |
| 2013 | 87 | 380 | 28 | 1.5 | 12.3 |
| 2014 | 100 | 940 | 50 | 0.89 | 15.0 |
| 2015 | 90 | 520 | 24 | 10.41 | 13.4 |
| 2016 | 87 | 590 | 25 | 2.89 | 13.4 |
| 2017 | 90 | 640 | 29 | 1.7 | 13.7 |
