# Supplementary material for: Immune-mediated hookworm clearance and survival of a marine mammal decrease with warmer ocean temperatures
Source: eLife. 2018 Nov 6;7:e38432. doi: 10.7554/eLife.38432 (PMC6245726; doi:10.7554/eLife.38432)
Supplement: Supplementary file 10. [file elife-38432-supp10.docx]

**Supplementary file 10**.

Regression models with hookworm prevalence, burden or mortality as response and sea surface temperature as predictor.

Hookworm prevalence

| Model | df | AIC | p-value | Ad-R^2^ |
| --- | --- | --- | --- | --- |
| Linear | 1 | 37.99704 | 0.06145 | 0.2928 |
| Parabolic | 2 | 38.84369 | 0.1316 | 0.2798 |
| Cubic | 3 | 39.68718 | 0.2144 | 0.2515 |

Hookworm burden

| Model | df | AIC | p-value | Ad-R^2^ |
| --- | --- | --- | --- | --- |
| Linear | 1 | 86.56169 | 6.05E-05 | 0.8645 |
| Parabolic | 2 | 88.39244 | 0.0005713 | 0.8478 |
| Cubic | 3 | 89.01772 | 0.002309 | 0.8452 |

Hookworm mortality

| Model | df | AIC | p-value | Ad-R^2^ |
| --- | --- | --- | --- | --- |
| Linear | 1 | 42.26951 | 0.01244 | 0.5082 |
| Parabolic | 2 | 44.26507 | 0.05517 | 0.4381 |
| Cubic | 3 | 45.96769 | 0.1376 | 0.3637 |
