# Supplementary material for: Immune-mediated hookworm clearance and survival of a marine mammal decrease with warmer ocean temperatures
Source: eLife. 2018 Nov 6;7:e38432. doi: 10.7554/eLife.38432 (PMC6245726; doi:10.7554/eLife.38432)
Supplement: Supplementary file 11. [file elife-38432-supp11.docx]

**Supplementary file 11**.

Regression models with hookworm prevalence, burden or mortality as response and chlorophyll-a mean concentrations as predictor.

Hookworm prevalence

| Model | df | AIC | p-value | Ad-R^2^ |
| --- | --- | --- | --- | --- |
| Linear | 1 | 38.79183 | 0.08871 | 0.2343 |
| Parabolic | 2 | 35.84365 | 0.04604 | 0.4665 |
| Cubic | 3 | 37.76831 | 0.1269 | 0.3822 |

Hookworm burden

| Model | df | AIC | p-value | Ad-R^2^ |
| --- | --- | --- | --- | --- |
| Linear | 1 | 105.681 | 0.2145 | 0.08331 |
| Parabolic | 2 | 103.7522 | 0.1235 | 0.2927 |
| Cubic | 3 | 105.5698 | 0.2649 | 0.1898 |

Hookworm mortality

| Model | df | AIC | p-value | Ad-R^2^ |
| --- | --- | --- | --- | --- |
| Linear | 1 | 44.15772 | 0.02819 | 0.4059 |
| Parabolic | 2 | 37.76683 | 0.005674 | 0.7066 |
| Cubic | 3 | 39.67652 | 0.02304 | 0.6608 |
