# Supplementary material for: Immune-mediated hookworm clearance and survival of a marine mammal decrease with warmer ocean temperatures
Source: eLife. 2018 Nov 6;7:e38432. doi: 10.7554/eLife.38432 (PMC6245726; doi:10.7554/eLife.38432)
Supplement: Supplementary file 12. [file elife-38432-supp12.docx]

**Supplementary file 12**.

Detail of sources, clone, retrieval methods and dilution of primary antibodies used for immunohistochemistry

| Antibody | Source | Antibody Clone, host species, antigen | Antigen Retrieval Method | Primary Antibody dilution | Visualization Method |
| --- | --- | --- | --- | --- | --- |
| CD3 | Dako^a^ | Monoclonal, mouse, Anti-human | Citrate | 1:1000 | DAB |
| CD21 | Cell Marque^b^ | Monoclonal, mouse, Anti-human | Reveal | 1:50 | DAB |
| Iba-1 | WAKO^c^ | Polyclonal, Rabbit, Anti-human | Citrate | 1:8000 | DAB |
| Mum1 | BioCare^d^ | Monoclonal, Rabbit, Anti-human | Citrate | 1:50 | DAB |
| C-kit (CD117) | Cell Marque^b^ | Monoclonal, Rabbit, Anti-human | Citrate | RTU | DAB |
| IL-4 | Mybiosource^e^ | Polyclonal, Rabbit, Anti-dog | Citrate | 1:8000 | DAB |

^a^Dako= Agilent Technologies®, Santa Clara, CA, USA. ^b^Cell Marque= Cell Marque biologicals, San Ramon, California USA. ^c^WAKO= Wako Chemicals®, Richmond, VA, USA. ^d^BioCare= Biocare Medical®, Pacheco, CA, USA. MyBiosource= Mybiosource Inc., San Diego, California, USA. RTU= Ready to use antibody (no dilution). DAB=diaminobenzidine
